# Detecting *Borrelia burgdorferi*–Triggered Seronegative Rheumatoid Arthritis Using [ ^68^Ga]Ga‐DOTA–Siglec‐9–Positron Emission Tomography/Computed Tomography

**DOI:** 10.1002/acr2.90116

**Published:** 2026-07-31

**Authors:** Prince Dadson, Heli Ylä‐Outinen, Simona Malaspina, Mikko Koivumäki, Sirpa Jalkanen, Laura Pirilä, Anne Roivainen

**Affiliations:** ^1^ Turku PET Centre, Turku University Hospital, University of Turku Turku Finland; ^2^ Department of Pulmonary Diseases, Turku University Hospital University of Turku Turku Finland; ^3^ InFLAMES Research Flagship Center University of Turku Turku Finland; ^4^ MediCity Research Laboratory University of Turku Turku Finland; ^5^ Centre for Rheumatology and Clinical Immunology, Division of Medicine Turku University Hospital, University of Turku Turku Finland

Lyme borreliosis is a tick‐borne infection caused by *Borrelia burgdorferi* that can involve the skin, joints, cardiovascular system, nervous system, and eyes.^1^ Infection with *B burgdorferi* is a recognized precursor to inflammatory arthritis and carries an increased long‐term risk of autoimmune joint disease, including rheumatoid arthritis (RA).^1^ A subset of patients develops persistent, symmetric polyarthritis that is clinically indistinguishable from seronegative RA despite successful eradication of the organism. Accurately distinguishing antibiotic‐refractory Lyme arthritis from true infection‐triggered autoimmunity remains one of the most consequential diagnostic challenges at the interface of infectious disease and rheumatology.

Persistent immune activation after *B burgdorferi* infection may result from molecular mimicry between outer surface protein A and host antigens, sustaining autoreactive T cell responses after pathogen clearance.^2^ Synovial tissue in antibiotic‐refractory Lyme arthritis demonstrates pannus formation and neoangiogenesis indistinguishable from RA.^3^ Ultrasound and 2‐deoxy‐2‐[^18^F]fluoro‐*D*‐glucose ([^18^F]FDG)–positron emission tomography/computed tomography (PET/CT) detect joint inflammation but cannot distinguish immune‐mediated synovitis from residual postinfectious inflammation. Gallium‐68‐labeled 1,4,7,10‐tetraazacyclododecane‐*N*,*N*′,*N*″,*N*′″‐tetraacetic acid‐conjugated sialic acid‐binding immunoglobulin‐like lectin 9 containing peptide ([^68^Ga]Ga‐DOTA–Siglec‐9)–PET/CT targets vascular adhesion protein 1 (VAP‐1), an endothelial molecule rapidly expressed on the cell surface in response to inflammation and central to leukocyte trafficking from the blood into tissues.^4^

We report, to our knowledge, the first case of *B burgdorferi*–triggered seronegative RA evaluated with [^68^Ga]Ga‐DOTA–Siglec‐9–PET/CT. A 77‐year‐old woman presented with rapidly progressive polyarthritis beginning in both knees and progressing within weeks to symmetric involvement of the wrists, metacarpophalangeal, proximal interphalangeal, hip, ankle, and metatarsophalangeal joints. C‐reactive protein levels were markedly elevated, and synovial fluid aspirated from the knee was inflammatory. Rheumatoid factor, anti–cyclic citrullinated peptide antibodies, antinuclear antibodies, and antineutrophil cytoplasmic antibodies were persistently negative. Serological testing subsequently demonstrated isolated *Borrelia* IgM positivity with negative IgG, consistent with recent or partially resolved infection.

Musculoskeletal ultrasound confirmed widespread synovial hypertrophy with pathologic power Doppler signal across more than 20 joints. [^18^F]FDG‐PET/CT demonstrated diffuse articular uptake in the wrists, metacarpophalangeal and proximal interphalangeal joints, shoulders, elbows, hips, knees, ankles, and metatarsophalangeal joints but could not distinguish autoimmune from postinfectious inflammation. In contrast, [^68^Ga]Ga‐DOTA–Siglec‐9–PET/CT revealed increased tracer accumulation across the same clinically affected joints (Figure 1). The pattern and intensity of [^68^Ga]Ga‐DOTA–Siglec‐9 uptake interpreted in the clinical context supported established autoimmune arthritis rather than persistent postinfectious synovitis. The diagnosis was seronegative RA (International Statistical Classification of Diseases and Related Health Problems, Tenth Revision: M06.0), with *B burgdorferi* infection identified as the putative triggering event.

**Figure 1:**
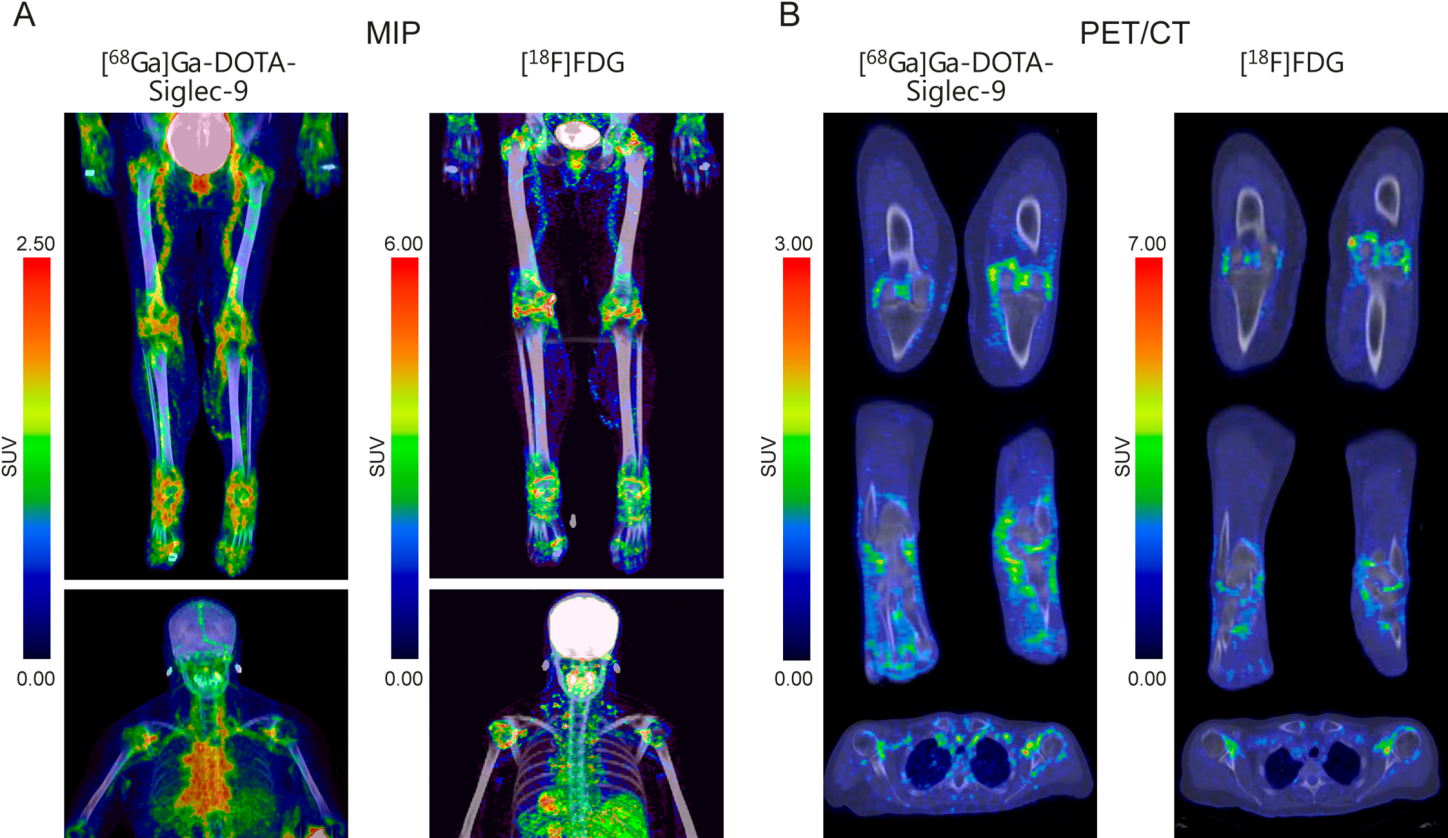
(A) Maximum intensity projection (MIP) images demonstrating increased bilateral tracer uptake in the knees, ankles, and metatarsophalangeal joints (upper panels) and shoulders (lower panels). (B) Representative fused positron emission tomography/computed tomography (PET/CT) cross‐sectional images of the knees (upper images), ankles and metacarpophalangeal joints (middle images), and shoulders (lower images), demonstrating increased [^68^Ga]Ga‐DOTA–Siglec‐9 uptake corresponding to clinically and ultrasonographically confirmed active synovitis. [^18^F]FDG, ^18^F‐labeled fluorodeoxyglucose; SUV, standardized uptake value.

This case highlights a clinically consequential transition from antibiotic‐refractory postinfectious synovitis to established autoimmune arthritis. Serological assays reflect immunologic memory rather than disease activity, whereas [^18^F]FDG‐PET/CT reflects increased glucose metabolism rather than immune cell–specific activity. Persistent [^68^Ga]Ga‐DOTA–Siglec‐9 uptake after antibiotic therapy indicated that inflammation was no longer infection driven but reflected autonomous immune activity, directly supporting escalation to disease‐modifying antirheumatic therapy.

Management of infection‐triggered inflammatory arthritis at the infectious–autoimmune interface lacks prospective trial evidence, and the risk of immunosuppression in inadequately treated infection must be weighed carefully against the morbidity of uncontrolled immune‐mediated joint destruction. In this setting, VAP‐1–targeted PET imaging may provide clinically decisive information. Radiation exposure with [^68^Ga]Ga‐DOTA–Siglec‐9–PET is substantially lower than with [^18^F]FDG‐PET,^4^ supporting its use in longitudinal assessment. Prospective studies are warranted to define its diagnostic performance across the spectrum of postinfectious arthritis and to validate its utility as a biomarker guiding therapeutic decisions.

## AUTHOR CONTRIBUTIONS

All authors contributed to at least one of the following manuscript preparation roles: conceptualization AND/OR methodology, software, investigation, formal analysis, data curation, visualization, and validation AND drafting or reviewing/editing the final draft. As corresponding author, Dr Roivainen confirms that all authors have provided the final approval of the version to be published and takes responsibility for the affirmations regarding article submission (eg, not under consideration by another journal), the integrity of the data presented, and the statements regarding compliance with institutional review board/Declaration of Helsinki requirements.

## Supporting information


**Disclosure form**.
